# Kinetic and quantitative analysis of [^18^F]SMBT-1 PET imaging for monoamine oxidase B

**DOI:** 10.1007/s12149-025-02083-y

**Published:** 2025-07-15

**Authors:** Kotaro Hiraoka, Berihu Mesfin, Yingying Wu, Yuki Shimizu, Asuka Kikuchi, Ryuichi Harada, Aiko Ishiki, Yoshihito Funaki, Shozo Furumoto, Shunji Mugikura, Nobuyuki Okamura, Akio Kikuchi, Kazuhiko Yanai, Hiroyuki Arai, Hiroshi Watabe, Manabu Tashiro

**Affiliations:** 1 Nuclear Medicine Laboratory, Research Center for Accelerator and Radioisotope Science, Tohoku University, Sendai, Japan; 2 Radiopharmaceutical Laboratory, Research Center for Accelerator and Radioisotope Science, Tohoku University, Sendai, Japan; 3 Department of Diagnostic Radiology, Tohoku University Hospital, Tohoku University, Sendai, Japan; 4 Division of Pharmacology, Faculty of Medicine, Tohoku Medical and Pharmaceutical University, Sendai, Japan; 5 Division of Geriatric and Community Medicine, Tohoku Medical and Pharmaceutical University, Sendai, Japan; 6 Department of Occupational Therapy, Yamagata Prefectural University of Health Sciences, Yamagata, Japan; 7 Department of Pharmacology, Tohoku University Graduate School of Medicine, Sendai, Japan; 8 Department of Geriatrics and Gerontology, Institute of Development, Aging and Cancer, Tohoku University, Sendai, Japan; 9 Radiation Protection and Safety Control Laboratory, Research Center for Accelerator and Radioisotope Science, Tohoku University, Sendai, Japan

**Keywords:** [^18^F]SMBT-1, PET, Monoamine oxidase B (MAO-B), Neuroinflammation

## Abstract

**Background and objective:**

In neuroinflammation, activated astrocytes, called reactive astrocytes, highly express monoamine oxidase B (MAO-B). [^18^F]SMBT-1 is a novel PET tracer developed for imaging neuroinflammation, with highly selective binding to MAO-B. The quantification method for [^18^F]SMBT-1 PET imaging has not been established, although some human studies using [^18^F]SMBT-1 PET imaging have already been conducted. In this study, we explored the most appropriate method for quantifying [^18^F]SMBT-1 PET.

**Methods:**

Dynamic PET scanning of [^18^F]SMBT-1, accompanied by serial arterial blood sampling, was performed in healthy elderly subjects. With PET and blood data, the total distribution volumes (Vts) in the brain regions were calculated using a one-tissue compartment model (1TCM), a two-tissue compartment model (2TCM), and Logan graphical analysis. Standardized uptake values (SUVs) and SUV ratio-1 (SUVR-1) were determined for different time frames and reference regions.

**Results:**

The values of the χ^2^ criterion and Akaike's Information Criterion (AIC) in the brain regions were lower in 2TCM than in 1TCM, suggesting that 2TCM was a better model in terms of curve fitting. However, the very high coefficient of variation (%COV) for parameters such as K1, k2, k3, and k4 in 2TCM suggests that these parameters may not have been properly estimated. SUVs, especially at 50–70 and 70–90 min post-injection, were strongly correlated with Vt (r = 0.9188–0.9445, p < 0.0001). SUVR-1 at these time points, referenced to various regions, showed significant correlations with MAO-B distribution in the brain shown in a previous postmortem study (r = 0.9362–0.9399, p < 0.0001).

**Conclusions:**

These findings suggest that SUVR-1, especially at 50–70 min and 70–90 min post-injection, reflects MAO-B distribution and is useful for quantifying [^18^F]SMBT-1 PET imaging, potentially enabling noninvasive assessment of neuroinflammation in the brain.

**Trial registration:**

Japan Registry of Clinical Trials (jRCT) (jRCTs021200019). It was registered on August 25, 2020. The jRCT was approved as a member of the Primary Registry Network of the WHO ICTRP.

**Supplementary Information:**

The online version contains supplementary material available at 10.1007/s12149-025-02083-y.

## Background

Neuroinflammation is a biological response of the central nervous system that removes injurious stimuli and initiates the healing process to protect cells and function of the central nervous system [1, 2]. Neuroinflammation is known to be involved not only in acute diseases such as stroke and encephalitis but also in chronic conditions such as Alzheimer's disease [2, 3]. The primary mediators of neuroinflammation are microglia, immune cells residing within the central nervous system [1]. Receiving immunological signals including cytokines, microglia are activated, and they start an activity such as phagocytosis of cell debris [2]. Astrocytes also receive immunological signals and are activated during neuroinflammation [2, 4]. Activated astrocytes, called “reactive astrocytes,” secrete inflammatory signals and neurotrophic and protective factors.Reactive astrocytes highly express monoamine oxidase B (MAO-B), an enzyme that catabolizes dopamine, in the outer mitochondrial membrane [5]. As reactive astrocytes highly express MAO-B in neuroinflammation, it can be a biomarker of neuroinflammation.

Various PET tracers have been developed for MAO-B imaging [6, 7]. ^11^C-L-deprenyl-D_2_ served as the primary PET tracer for visualizing MAO-B in the human brain. However, irreversible binding complicates the quantification process. To overcome this issue, researchers developed ^11^C-SL25.1188, a reversible MAO-B PET tracer that has been utilized in human studies [8]. The short 20-min half-life of ^11^C limits the clinical application of PET tracers. Consequently, several ^18^F-labeled PET tracers have been developed to facilitate MAO-B imaging [9–12]. [^18^F]SMBT-1 is a PET tracer for MAO-B imaging that was recently developed by Harada et al. [13]. In vitro binding assays and autoradiographic analysis showed that [^18^F]SMBT-1 exhibits highly selective binding to MAO-B. Furthermore, an animal study using mice showed high uptake in the brain, rapid washout, and no radiolabeled metabolites in the brain. Moreover, [^18^F]SMBT-1 has been evaluated in human subjects and showed higher binding in the brains of patients with Alzheimer’s disease than in amyloid-negative cognitively unimpaired subjects, which reflects neuroinflammation in the brains of patients with Alzheimer’s disease [14, 15].

To accurately interpret PET data, it is essential to develop a quantitative approach. There are various quantification methods, such as compartment model analysis, Logan graphical method, and the ratio of standardized uptake values between the region of interest and reference region (SUVR) [16]. An ideal quantitative approach would accurately represent the distribution of MAO-B in brain regions without the need for invasive techniques, such as blood sampling. In studies by Villemagne et al. [14, 15], an SUVR of 60–80 min after [^18^F]SMBT-1 administration referenced to the subcortical white matter was adopted for quantification. In another study on the kinetic analysis of [^18^F]SMBT-1, SUVR at 70–90 min post-injection, referenced to the cerebellar gray matter, was used for quantification [17]. The value of SUVR varies depending on which time frame is selected and which region is chosen as the reference region, but neither study has thoroughly examined which combination of time frame and reference region best reflects the density of MAO-B in the brain region. Thus, there are various quantification options, and a quantitative method for [^18^F]SMBT-1 PET has not yet been established.

In this study, we performed 90 min [^18^F]SMBT-1 PET scanning with arterial blood sampling in healthy human subjects and compared different quantification methods. Using PET and blood data, we calculated the total distribution volume (Vt) in the brain regions using a compartment model analysis and the Logan graphical method. We investigated the correlation between the distribution volumes and standardized uptake values (SUVs) in different time frames. SUVR-1 is thought to be more appropriate than SUVR as an index of MAO-B distribution in the tissues. We investigated the correlations between SUVR-1 in different time frames and referenced to different regions and MAO-B distribution in brain regions measured in a preceding postmortem study [18] to determine which method of calculating SUVRs might be adequate for quantifying MAO-B in the brain.

## Materials and methods

### Subjects

Six healthy participants were enrolled in this study. Four of the patients were female and two were male. The age of the subjects was 73.7 ± 8.4 (mean ± standard deviation). They had no cognitive impairment, neurological diseases, or apparent abnormalities on brain magnetic resonance (MR) images. The patients did not receive any centrally acting medications. This study was approved by The Ethics Committee of Tohoku University School of Medicine, registered in jRCT (jRCTs021200019), and conducted in accordance with the 1964 Declaration of Helsinki and all subsequent revisions. Written informed consent was obtained from all subjects.

### PET scanning, blood sampling, metabolite analysis, and magnetic resonance imaging

The PET scanner used in this study was an Eminence SET-3000B/X scanner (Shimadzu Corp., Kyoto, Japan). [^18^F]SMBT-1 was injected intravenously into each subject, and a 90-min dynamic scan with 29 time frames (10 s × 12 frames, 1 min × 2 frames, 2 min × 1 frame, 4 min × 1 frame, 5 min × 10 frames, and 10 min × 3 frames) was performed. The injected dose of [^18^F]SMBT-1 was 188.4 ± 32.5 MBq (mean ± standard deviation). During PET scanning, 1 mL of arterial blood was sampled 29 times from the radial artery to measure plasma activity, and an additional 1.5 mL of arterial blood was sampled at approximately 5, 15, 30, 60, and 90 min after [^18^F]SMBT-1 injection to measure the metabolite fraction of [^18^F]SMBT-1. For metabolite analysis, a 0.5 mL plasma sample was added to a tube containing 0.75 mL acetonitrile and mixed vigorously for 2 min. After centrifugation (14,000 × g, 5 min), the resulting supernatant was diluted with 0.6 mL of phosphate-buffered saline (PBS) containing 25 μg of cold SMBT-1 and passed through a 0.22-μm membrane filter. The filtrate was injected into an HPLC system (Column: InertSustain C18 column, 7.6 × 150 mm and 5 μm; GL Sciences, Inc.) with a PREP guard column (7.6 mm × 30 mm; GL Sciences, Inc.) eluted with a mixture of 20 mM NaH_2_PO_4_: acetonitrile (60:40) at a flow rate of 2 mL/min. The eluate was collected in two fractions: fraction A collected from the time of injection to the time of elution of SMBT-1 and fraction B collected during the elution of SMBT-1 (as detected by UV 340 nm). The fractions were counted using a gamma counter (AccuFlex γ7000; ALOKA, Tokyo, Japan). The fraction of parent [^18^F]SMBT-1 was determined by dividing the counts in fraction B by the total counts in fractions A and B, as previously described [19]. For anatomical reference in PET image analysis, 3D MR images of each subject were acquired using an MR scanner (Ingenia CX, Philips Medical Systems).

### PET image analysis

The PKIN and PNEURO tools in the PET image analysis software PMOD (version 4.3, PMOD Technologies Ltd., Zurich, Switzerland) [20] were used for the image analysis. Dynamic PET and MR data were loaded into the PNEURO tool to obtain time-activity curves in brain tissues (tTACs). The individual gray matter probability map was calculated by segmentation of the MR images. PET images of each subject were rigidly matched to individual MR images. MR images were spatially normalized to the Montreal Neurological Institute (MNI) T1 template. The VOI (volume of interest) atlas was transformed into MR space, and the cortical structures with the gray matter probability map were intersected. The VOIs (frontal, parietal, temporal, occipital, cingulate, and insular cortices, pallidum, thalamus, caudate, putamen, and amygdala) were applied to the matched PET series to calculate the tTACs.

As the original plasma time-activity curve (pTAC) values were the sum of the activities of unmetabolized [^18^F]SMBT-1 and metabolized [^18^F]SMBT-1, the original pTAC values were corrected, and metabolite-corrected pTAC values were obtained. For this correction, the empirical function proposed by Watabe et al. to express the fraction of the unmetabolized tracer at time *t* [21], 1 / (1 + (αt)^2^)^β^, was fitted using a least-squares method in GraphPad PRISM 5 (GraphPad Software, Inc.).

Subsequently, the tTACs of ROIs (regions of interest) and metabolite-corrected pTACs were loaded into the PKIN tool. For PKIN, a one-tissue compartment model (1TCM), two-tissue compartment model (2TCM), and Logan graphical analysis were applied to calculate the parameters and quantify PET data [16, 22]. In 1TCM, K1 is the rate constant of transference from plasma to tissue through the blood–brain barrier (BBB), and k2 denotes the back-diffusion from tissue to plasma. In the 2TCM, based on the assumption that the non-specific binding component is in equilibrium with the free component [23], K1 is the rate constant of transfer from plasma to tissue through the BBB, k2 is the rate constant of back-diffusion from tissue to plasma, and k3 and k4 denote the association and dissociation rate constants, respectively, between the free and specifically bound components. The rate constants were estimated nonlinearly using the Levenberg–Marquardt algorithm. The blood volume (vB) was fixed at 3% [24], and initial values were 0.5 for K1, 0.035 for k2, 0.01 for k3, and 0.01 for k4. Vt was calculated as K1/k2 in the 1TCM and (K1/k2) (1 + k3/k4) in the 2TCM [25, 26]. For the comparison of 1TCM and 2TCM, goodness of fit was evaluated using the χ^2^ criterion and model relevance was assessed using Akaike's Information Criterion (AIC) [27]. In the Logan graphical analysis, the starting time of the linear section in the graphical plot was fitted using an error criterion of 10%.

To calculate the SUVs of brain regions and reference regions in different time frames, the averaged SUV images from 30 to 50 min, 50 to 70 min, and 70 to 90 min after [^18^F] SMBT-1 injection were produced from dynamic PET data in PMOD. The averaged SUV images and MR data were loaded into the PNEURO tool, and the SUVs of the ROIs (frontal, parietal, temporal, occipital, cingulate, and insular cortices, pallidum, thalamus, caudate, putamen, and amygdala) and reference regions were calculated. The cerebral deep white matter, subcortical white matter, cerebellar cortex, and cerebellar white matter were used as reference regions. SUVR-1 of the brain regions was calculated by subtracting one from the ratio between the SUVs of the brain regions and the reference regions.

### Statistical analysis

Correlations between Vts and SUVs and between SUVR-1 and MAO-B distribution in the brain regions shown in the preceding postmortem study [18] were analyzed by Pearson’s correlation analysis using GraphPad PRISM 10 (GraphPad Software, Inc.). To investigate the correlation between SUVR-1 and MAO-B distribution in different brain regions, data from a previous postmortem study [18] were used. In the postmortem study, the specific binding of [^11^C]-l-deprenyl, the radioligand version of l-deprenyl (selegiline), a selective irreversible MAO-B inhibitor, was investigated in the brain without pathology using autoradiography.

## Results

### Time activity curves of plasma and brain regions (pTACs and tTACs)

**Fig. 1:**
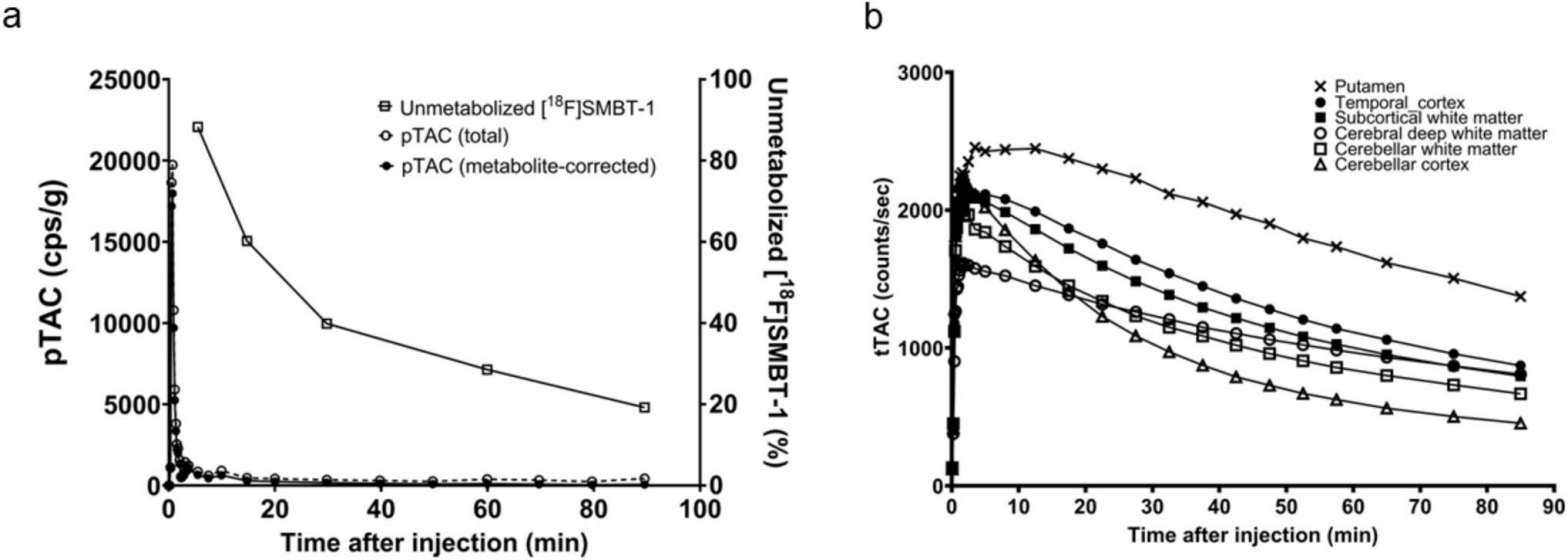
Representative plasma time-activity curve (pTAC), fraction of unmetabolized [^18^F]SMBT-1 in arterial plasma, and tissue time-activity curves (tTACs) of brain regions (Subject 1). **a** The total and metabolite-corrected radioactivity are plotted as dashed and solid lines, respectively. The amount of unmetabolized [^18^F]SMBT-1 is shown in the upper curve. At 30 min, 40% of the administered [^18^F]SMBT-1 remained in its unmetabolized form. **b** tTACs of the putamen, temporal cortex, subcortical white matter, deep cerebral white matter, cerebellar white matter, and cerebellar cortex

PET data were obtained from all six subjects; however, blood samples were obtained from three of them. Blood samples could not be collected from the other three subjects because arterial catheter insertion was unsuccessful owing to technical issues. The total and metabolite-corrected pTAC and changes in the unmetabolized [^18^F]SMBT-1 fractions are shown in Fig. 1-a and Supplementary Fig. 1. Plasma activity rapidly increased and decreased after [^18^F]SMBT-1 injection, and the fraction of unmetabolized [^18^F]SMBT-1 gradually decreased to approximately 20% 90 min post-injection. The tTACs of the brain regions are shown in Fig. 1-b and Supplementary Fig. 1. tTACs rapidly increased after [^18^F]SMBT-1 injection and gradually decreased thereafter.

### Compartment model and Logan graphical analysis

Vts and kinetic parameters evaluated by the 1TCM and 2TCM analyses are shown in Table 1. The 1TCM parameters K1, k2, and Vt were well identified in all regions, with mean coefficient of variation (%COV) values of 12.90%, 3.57%, and 11.82% respectively. The 2TCM microparameters K1, k2, k3, and k4 were poorly identified in all regions, with mean %COV values of 44.23%, 93.95%, 86.16%, and 27.84%, respectively. The 2TCM macroparameter Vt was well-identified in all regions, with a mean %COV value of 6.57%.

**Table 1:** Kinetic parameters estimated by the 1TCM and the 2TCM (mean and %COV, n = 3)

	K1 (ml/ccm/min)		k2 (/min)		Vt (ml/ccm)					
	Mean	%COV	Mean	%COV	Mean	%COV				
1TCM										
Cerebral deep white matter	0.16	14.61	0.03	1.40	4.81	13.68				
Pallidum	0.19	14.82	0.02	6.50	7.82	8.86				
Caudate	0.17	11.42	0.02	7.37	8.08	6.12				
Thalamus	0.25	16.78	0.03	4.88	7.91	15.56				
Cingulate cortex	0.26	15.23	0.04	2.96	6.30	13.13				
Insular cortex	0.23	13.86	0.04	2.58	6.19	11.89				
Parietal cortex	0.23	12.99	0.05	2.81	4.75	12.75				
Amygdala	0.20	11.72	0.02	0.85	8.94	12.02				
Temporal cortex	0.21	9.61	0.04	1.65	5.37	8.61				
Frontal cortex	0.23	14.30	0.04	2.77	5.04	12.88				
Occipital cortex	0.23	8.71	0.05	2.31	4.23	10.82				
Putamen	0.23	12.35	0.03	4.67	8.40	9.62				
Cerebellar cortex	0.24	11.00	0.07	7.77	3.56	15.28				
Cerebellar white matter	0.21	12.33	0.05	3.42	4.25	13.77				
Subcortical white matter	0.22	13.70	0.04	1.56	5.07	12.24				

	K1 (ml/ccm/min)		k2 (/min)		k3 (/min)		k4 (/min)		Vt (ml/ccm)	
	Mean	%COV	Mean	%COV	Mean	%COV	Mean	%COV	Mean	%COV
2TCM										
Cerebral deep white matter	0.20	15.83	0.19	29.42	0.21	44.15	0.04	30.81	5.66	6.66
Pallidum	0.39	65.49	2.75	134.48	1.01	117.7	0.03	24.78	9.20	2.41
Caudate	0.22	18.14	0.32	71.03	0.65	84.94	0.05	22.78	9.02	0.55
Thalamus	0.54	65.68	2.85	127.78	1.10	100.96	0.04	21.49	8.68	10.41
Cingulate cortex	0.56	64.42	2.89	125.21	1.13	95.67	0.06	24.45	6.77	8.82
Insular cortex	0.49	62.65	2.88	126.05	1.16	99.20	0.05	22.02	6.71	6.96
Parietal cortex	0.47	62.08	2.86	127.08	1.11	103.84	0.06	29.12	5.11	8.11
Amygdala	0.24	13.72	0.17	37.9	0.28	54.35	0.04	25.57	10.6	7.76
Temporal cortex	0.42	60.48	2.83	128.95	1.15	106.29	0.05	24.30	5.83	3.81
Frontal cortex	0.48	63.86	2.87	126.46	1.09	101.44	0.06	25.75	5.47	8.08
Occipital cortex	0.46	57.67	2.85	127.79	1.11	107.73	0.07	28.52	4.56	6.21
Putamen	0.49	64.45	2.79	131.82	1.10	106.97	0.04	27.66	9.28	4.32
Cerebellar cortex	0.33	18.93	0.50	46.33	0.58	70.41	0.11	41.94	3.74	12.24
Cerebellar white matter	0.28	14.62	0.31	25.98	0.27	42.34	0.06	30.14	4.79	8.42
Subcortical white matter	0.27	15.45	0.22	43.02	0.25	56.43	0.07	38.28	5.45	3.74

1TCM, one-tissue compartment model; 2TCM, two-tissue compartment model; Vt, total distribution volume; %COV, coefficient of variation of the parameter shown as a percentage of parameter value

Supplementary Fig. 2 shows representative images of the curve fittings of 1TCM and 2TCM. The curve fits better to actual tTAC in 2TCM compared to 1TCM. The suitability of the curve fittings for 1TCM and 2TCM was evaluated using the χ^2^ criterion and Akaike’s information criterion (AIC) (Supplementary Table 1). The values of the χ^2^ criterion and AIC in brain regions were lower in 2TCM than in 1TCM, suggesting that 2TCM is more appropriate for [^18^F]SMBT-1 than 1TCM in terms of curve fitting. A representative Logan plot and Vts evaluated by Logan graphical analysis are shown in Supplementary Fig. 3 and Supplementary Table 2.

The Vts estimated by 1TCM, 2TCM, and Logan graphical analysis showed significant correlations (Supplementary Fig. 4), which varidates the Vts each other.

### Correlation between SUVs and Vts

**Fig. 2:**
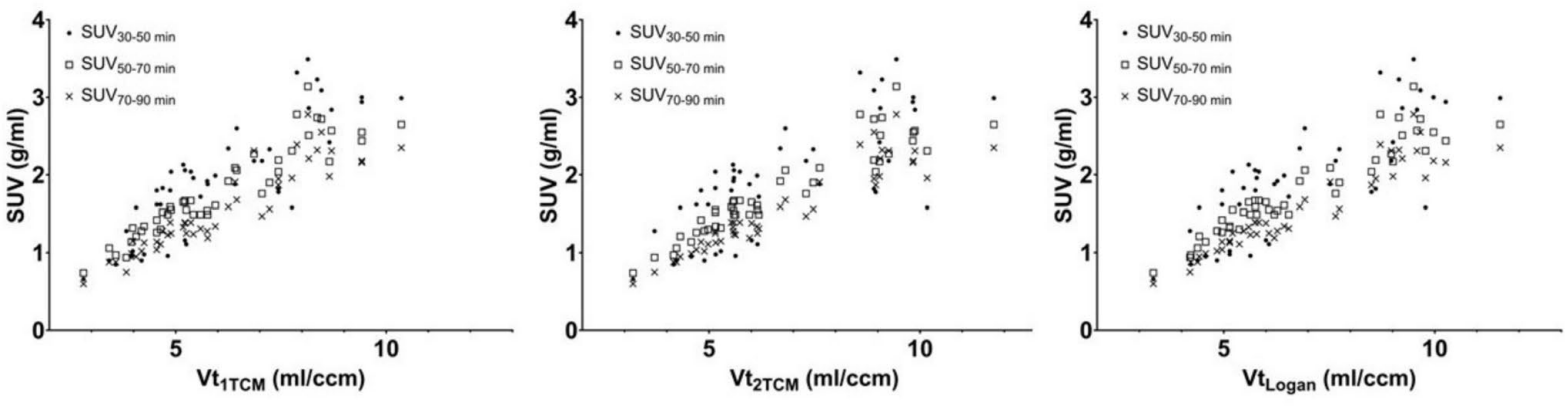
Correlation between Vts estimated by 1TCM, 2TCM, and Logan graphical analysis (Vt_1TCM_, Vt_2TCM_, Vt_Logan_) and SUVs in the time frames–30–50 min, 50–70 min, and 70–90 min after [^18^F]SMBT-1 injection (SUV_30-50 min_, SUV_50-70 min_, and SUV_70-90 min_) (n = 3). The data of all brain regions are plotted. Vt, total distribution volume; 1TCM, a one-tissue compartment model; 2TCM, a two-tissue compartment model; SUV, standardized uptake value

The means and standard deviations of the SUVs in the brain regions at different time frames are shown in Supplementary Table 3. SUVs at 30–50, 50–70, and 70–90 min after [^18^F]SMBT-1 injection showed a significant correlation with Vts estimated by 1TCM, 2TCM, and Logan graphical analysis (p < 0.0001) (Fig. 2 and Table 2). Pearson’s r of SUVs at 30–50 min post-injection was lower than that of SUVs at 50–70 min and 70–90 min.

**Table 2:** Correlation between SUV in different time frames and Vts estimated by 1TCM, 2TCM, and Logan graphical analysis (n = 3)

	SUV_30-50 min_	SUV_50-70 min_	SUV_70-90 min_
Vt_1TCM_			
Pearson r	0.8483	0.9280	0.9188
P value	< 0.0001	< 0.0001	< 0.0001
Vt_2TCM_			
Pearson r	0.7582	0.9314	0.9388
P value	< 0.0001	< 0.0001	< 0.0001
Vt_Logan_			
Pearson r	0.7999	0.9396	0.9445
P value	< 0.0001	< 0.0001	< 0.0001

SUV_30-50_, SUV_50-70_, and SUV_70-90 min_, SUVs in the time frames–30–50 min, 50–70 min, and 70–90 min after [^18^F]SMBT-1 injection; 1TCM, a one-tissue compartment model; 2TCM, a two-tissue compartment model; Vt_1TCM_, Vt_2TCM_, Vt_Logan,_ Vt estimated by 1TCM, 2TCM, and Logan graphical analysis

### Correlation between SUVR-1 in different time frames and referenced to different regions and MAO-B distribution in brain regions shown in the preceding postmortem study

**Fig. 3:**
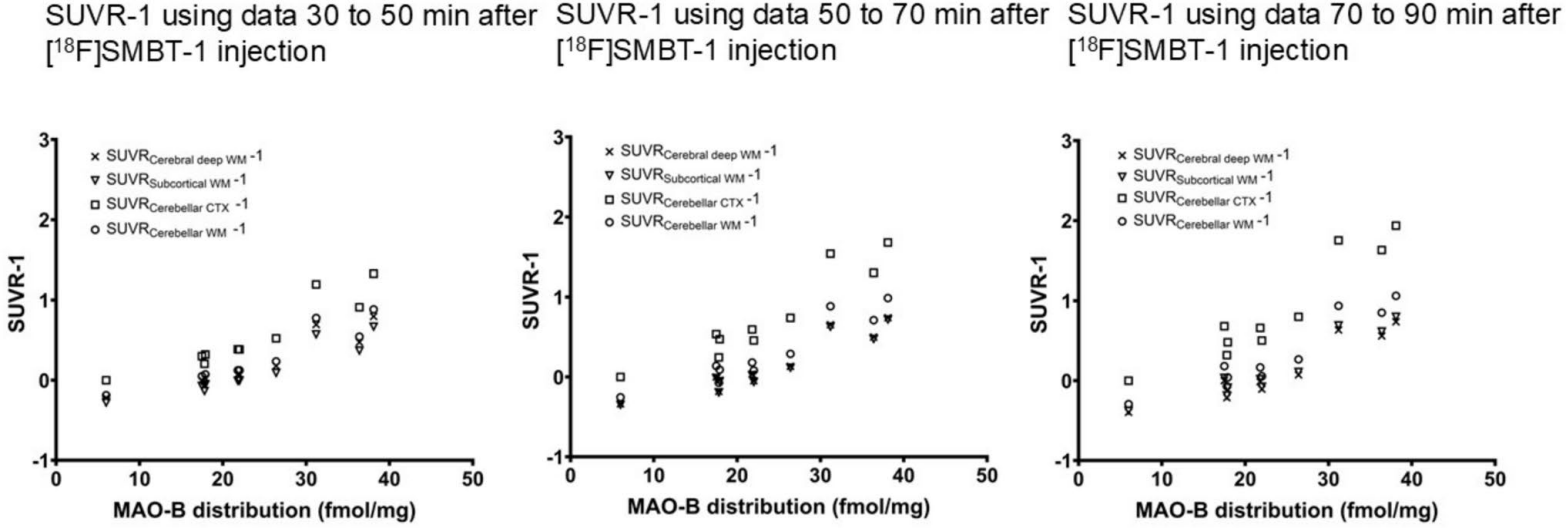
The correlation between the mean SUVR-1 of six subjects in each brain region and MAO-B distribution in the brain shown in a previous postmortem study [18]. SUVR, standardized uptake value ratio; SUVR_Cerebral deep WM_, SUVR referenced cerebral deep white matter; SUVR_Subcortical WM_, SUVR referenced subcortical white matter; SUVR_Cerebellar CTX_, SUVR referenced cerebellar cortex; SUVR_Cerebellar WM_, SUVR referenced cerebellar white matter; MAO-B, monoamine oxidase B

The mean and standard deviation of SUVR-1 in the brain regions at different time frames are shown in Supplementary Table 3. All SUVR-1 at different time frames and referenced to different regions showed significant correlations with MAO-B distribution in brain regions in a previous postmortem study [18] (p < 0.0001) (Fig. 3 and Table 3). The Pearson’s r of SUVR-1 at 30–50 min post-injection (0.9248–0.9257) was lower than that of SUVR-1 at 50–70 min and 70–90 min (0.9362–0.9399).

**Table 3:** Correlation between SUVR-1 in different time frames and MAO-B distribution in the brain (n = 6)

Reference region	Cerebral deep WM	Subcortical WM	Cerebellar CTX	Cerebellar WM
Time frame: 30–50 min after [^18^F]SMBT-1 injection				
Pearson r	0.9248	0.9252	0.9257	0.9251
P value	0.0001	0.0001	0.0001	0.0001
Time frame: 50–70 min after [^18^F]SMBT-1 injection				
Pearson r	0.9363	0.9362	0.9371	0.9370
P value	< 0.0001	< 0.0001	< 0.0001	< 0.0001
Time frame: 70–90 min after [^18^F]SMBT-1 injection				
Pearson r	0.9387	0.9387	0.9399	0.9394
P value	< 0.0001	< 0.0001	< 0.0001	< 0.0001

SUVR, standardized uptake value ratio; WM, white matter; CTX, cortex

## Discussion

In this study, the kinetics of [^18^F]SMBT-1 were analyzed and various quantification methods for [^18^F]SMBT-1 PET data were examined. The rapid increase and gradual decrease in pTAC after [^18^F]SMBT-1 injection indicates fast delivery and diffusion of [^18^F]SMBT-1 to body organs, including the brain. The chronological decrease in the unmetabolized [^18^F]SMBT-1 fraction suggests the gradual metabolization of [^18^F]SMBT-1. The rate of the unmetabolized fraction of [^18^F]SMBT-1 in Japanese individuals did not differ from that in Caucasians, as previously described [17]. Preclinical studies have identified cytosolic sulfotransferase 1A1 (SULT1A1) as the dominant metabolic enzyme in [^18^F]SMBT-1 [28]. Genetic polymorphism has been reported in SULT1A1. SULT1A1*2 is a genetic variant of SULT1A1. It is defined by an Arg213His polymorphism, which means that there is a change from arginine to histidine at position 213 in the protein sequence. SULT1A1*2 had significantly lower enzymatic activity than wild-type SULT1A1*1. The allele frequency of SULT1A1*2 is approximately 30% in Caucasians and 16.8% in the Japanese population [29–31]. In our study, the genetic polymorphisms in the subjects were not investigated. Although genetic polymorphisms did not influence the metabolism of [^18^F]SMBT-1 in an in vitro study [28], it is necessary to explore how these genetic variations might affect the metabolism of [^18^F]SMBT-1 in vivo in future research. The rapid increase and gradual decrease in tTACs in the brain regions suggest that [^18^F]SMBT-1 is rapidly distributed to brain tissues and later gradually released into the blood flow.

To estimate Vts, 1TCM, 2TCM, and Logan graphical methods were applied. In the compartment model analysis, we fixed the blood volume (vB) at 3% to reduce the degree of freedom and stabilize the estimation. However, if vB was fixed at a different value or was not fixed at all, the results of the parameter estimation would be different. We compared the results of parameter estimation (a part of the results is shown in Supplementary Table 4). In the 1TCM, Vt tended to be slightly larger when vB was fixed at 5% or not fixed compared to when vB was fixed at 3%. In 2TCM, when vB was not fixed, extremely high Vt was observed in some brain regions (e. g., Vt of the frontal cortex in Supplementary Table 4), and it seems that appropriate estimation of Vt was not possible. In the case of 2TCM with vB fixed at 5%, an extremely high Vt was observed in one brain region (data not shown). To examine the suitability of curve fittings, the values of χ^2^ criterion and AIC were calculated in 1TCM and 2TCM, respectively. These values were lower in 2TCM than in 1TCM, suggesting that 2TCM is more appropriate for [^18^F]SMBT-1 than 1TCM in terms of curve fitting. The 1TCM parameters K1, k2, and Vt were well identified in all regions, with a low %COV. Although the very high %COV for microparameters K1, k2, k3, and k4 in 2TCM suggests that these parameters may not have been properly estimated, the mean %COV of macroparameter Vt is low, suggesting that Vt is estimated properly in 2TCM. A significant correlation was observed between Vts and SUVs, suggesting that SUVs can be used as substitutions for Vts in the quantification of PET data. Pearson correlation coefficients of SUVs at 50–70 min post-injection and 70–90 min post-injection were higher than those of SUVs at 30–50 min post-injection, suggesting that SUVs later than 50 min post-injection reflect Vts.

For quantification of PET data, SUVR-1 were calculated. The strong correlation between Vts and SUV suggests that SUVR-1 approximates the binding potential (BP_ND_), which refers to the ratio at equilibrium of a specifically bound radioligand to that of a non-displaceable radioligand in tissue [25]. Therefore, we assumed that SUVR-1 was more appropriate than SUVR as an index of MAO-B distribution in tissues. Ideally, a brain region lacking MAO-B expression would serve as the best reference region. However, no such brain region was identified. Therefore, as an alternative, we selected brain regions with a minimal MAO-B distribution as potential reference regions. Autoradiographic experiments using [^11^C]-L-deprenyl [18] showed a lower MAO-B distribution in the cerebellar cortex than in other brain regions in the human brain without pathology. An immunohistochemical study of the normal aging human brain [32] showed the cerebellum has the lowest MAO-B expression level, especially the cerebellar cortex, among the brain regions. In a blocking study using oral administration of Selegiline, an MAO-B inhibitor, and [^18^F]SMBT-1 PET, the percentage reduction in regional [^18^F]SMBT-1 SUV was lower in the subcortical and cerebellar white matter than in other brain regions, suggesting that the proportion of specific binding to MAO-B to non-specific binding and free tracers is lower in these regions [14]. Therefore, we chose the cerebellar cortex and white matter, subcortical white matter, and cerebral deep white matter as reference regions.

To calculate SUVR-1, average SUV images at 30–50 min, 50–70 min, and 70–90 min post-injection were used, and each candidate reference region was applied. SUVR-1 at all time frames and all reference regions showed a significant correlation with MAO-B distribution in the brain regions shown in the preceding postmortem study. However, the Pearson correlation coefficients of SUVR-1 at 50–70 min and 70–90 min post-injection were higher than those at SUVR-1 at 30–50 min post-injection, suggesting that SUVR-1 later than 50 min post-injection might reflect MAO-B distribution.

For the reference region, the subcortical white matter was adopted in the study by Villemagne et al. [14], as the proportion of specific binding to MAO-B to non-specific binding and free tracers was lowest in the region investigated in a blocking study. In the study, SUVR referenced to the subcortical white matter showed a high correlation coefficient with MAO-B distribution in the brain regions shown in the preceding postmortem study. In a study by Lopresti et al. [17], the cerebellar cortex was adopted as the reference region because it showed low Vts estimated by 2TCM and the SUVR referenced to the cerebellar cortex showed a high correlation with the distribution volume ratio estimated by 2TCM. In our study, selection of the reference region did not significantly affect the correlation coefficients. The results indicate that several brain regions can be selected as reference regions, depending on the disease under study. The lowest MAO-B distribution in the cerebellar cortex among the brain regions was observed in postmortem studies [18, 32], and the MAO-B distribution was not significantly different between the cerebellar cortex in the brain with neuropathological changes of Alzheimer’s disease and the brain with no or few neuropathological changes of Alzheimer’s disease [32]. This suggests that the cerebellar cortex may be the most stable and adequate reference region for calculating SUVRs in Alzheimer’s disease. In the case of diseases with severe pathological conditions in the cerebellum, such as multiple system atrophy, it may be necessary to consider regions other than the cerebellum, such as the cerebral white matter, as reference regions.

This study has several limitations. First, the sample size was small, with PET data collected from six participants; however, blood samples were obtained from only three of them. This limited number of subjects might have skewed the results or obscured the differences that a larger sample could have revealed. Another limitation is the inability to ascertain the true density of MAO-B in each participant's brain, which means that we cannot confirm that the quantitative [^18^F]SMBT-1 PET values accurately represent MAO-B density. To address this, the study correlated these quantitative values with MAO-B density in various brain regions, as observed in autopsy studies of healthy individuals, to support their validity. Despite these constraints, this study may still provide valuable foundational data for clinical research involving [^18^F]SMBT-1 PET, particularly in quantitative analyses.

## Conclusions

The results of this study suggest that SUVR-1, particularly at 50–70 and 70–90 min post-injection, correlate well with the known MAO-B distribution in the brain. There is no ideal reference region without specific binding; however, the reference region should be carefully selected depending on the disease of interest for PET imaging. These findings indicate that SUVR-1 analysis may be a useful and practical approach for quantifying [^18^F]SMBT-1 PET data to assess MAO-B levels in the brain, and consequently, neuroinflammation. Although further validation is needed in larger cohorts, this study provides a foundation for optimizing [^18^F]SMBT-1 PET quantification methods to enable their use as noninvasive biomarkers of neuroinflammation in neurological disorders. The ability to reliably measure MAO-B with PET could significantly advance our understanding of the neuroinflammatory processes in the living human brain.

## Supplementary Information

Below is the link to the electronic supplementary material.

Supplementary file1 (DOCX 784 KB)

## Data Availability

The datasets generated during and/or analyzed during the current study are available from the corresponding author upon reasonable request.
