# Supplementary material for: Kinetic and quantitative analysis of [18F]SMBT-1 PET imaging for monoamine oxidase B
Source: Ann Nucl Med. 2025 Jul 15;39(11):1249–57. doi: 10.1007/s12149-025-02083-y (PMC12559039; doi:10.1007/s12149-025-02083-y)
Supplement: Supplementary file 1 — Supplementary file1 (DOCX 784 KB) [file 12149_2025_2083_MOESM1_ESM.docx]

**Supplementary figures and tables**

**Article title:** Kinetic and Quantitative Analysis of [^18^F]SMBT-1 PET Imaging for Monoamine Oxidase B

**Journal name:** Annals of Nuclear Medicine

**Author names:**

Kotaro Hiraoka^1,3^, Berihu Mesfin^1^, Yingying Wu^1^, Yuki Shimizu^2^, Asuka Kikuchi^1,3^, Ryuichi Harada^4^, Aiko Ishiki^5^, Yoshihito Funaki^2^, Shozo Furumoto^2,3^, Shunji Mugikura^3^, Nobuyuki Okamura^4^, Akio Kikuchi^6^, Kazuhiko Yanai^7^, Hiroyuki Arai^8^, Hiroshi Watabe^9,3^, Manabu Tashiro^1,3^

**Affiliation:**

^1^Nuclear Medicine Laboratory, Research Center for Accelerator and Radioisotope Science, Tohoku University, Sendai, Japan.

^2^Radiopharmaceutical Laboratory, Research Center for Accelerator and Radioisotope Science, Tohoku University, Sendai, Japan.

^3^Department of Diagnostic Radiology, Tohoku University Hospital, Tohoku University, Sendai, Japan

^4^Division of Pharmacology, Faculty of Medicine, Tohoku Medical and Pharmaceutical University, Sendai, Japan.

^5^Division of Geriatric and Community Medicine, Tohoku Medical and Pharmaceutical University, Sendai, Japan.

^6^Department of Occupational Therapy, Yamagata Prefectural University of Health Sciences, Yamagata, Japan.

^7^Department of Pharmacology, Tohoku University Graduate School of Medicine, Sendai, Japan.

^8^Department of Geriatrics and Gerontology, Institute of Development, Aging and Cancer, Tohoku University, Sendai, Japan.

^9^Radiation Protection and Safety Control Laboratory, Research Center for Accelerator and Radioisotope Science, Tohoku University, Sendai, Japan.

**e-mail address of corresponding author:** khiraoka@raris.tohoku.ac.jp

**Supplementary Fig. 1** Plasma time-activity curves, fractions of unmetabolized [^18^F]SMBT-1, and tissue time-activity curves of brain regions (the temporal cortex of Subject 2 and 3). pTAC, plasma time-activity curve; tTAC, tissue time-activity curve


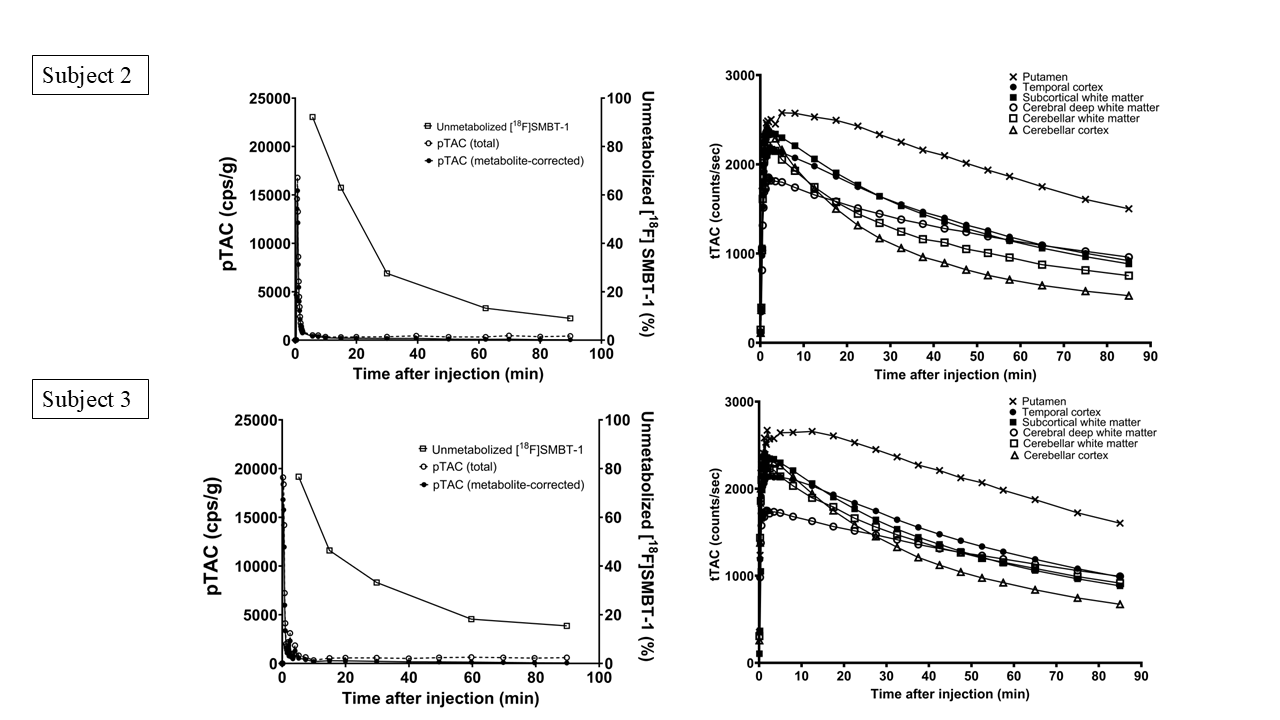


**Supplementary Fig. 2** Representative curve fittings by 1TCM and 2TCM (temporal cortex of Subject 1). The dots and lines represent the measured tissue time-activity curves and model fit, respectively. 1TCM, a one-tissue compartment model; 2TCM, a two-tissue compartment model; Vt, total distribution volume; AIC, Akaike information criterion.

**
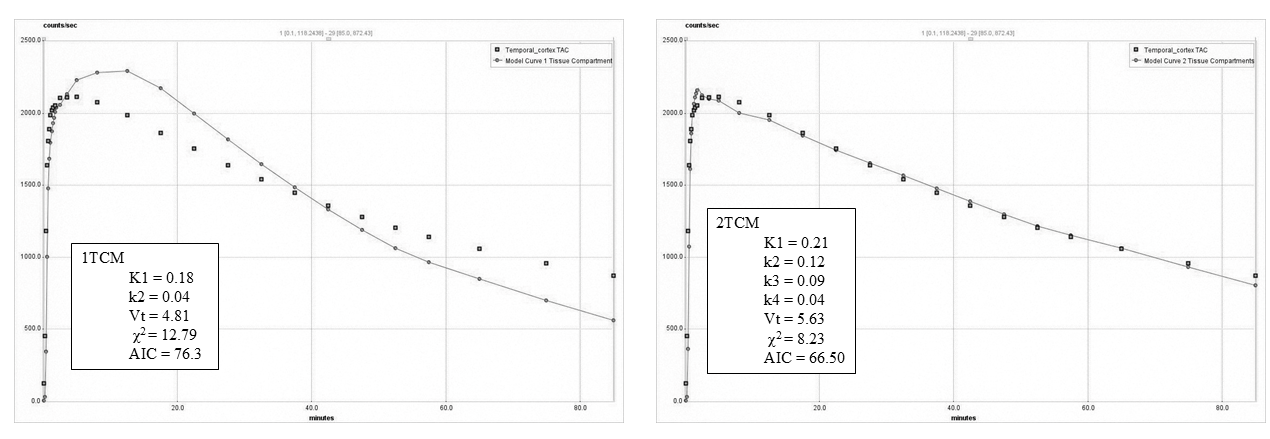
**

**Supplementary Fig. 3** Representative Logan plot (temporal cortex of Subject 1). C_Tissue and C_plasma represent the measured time-course of radioactivity in the tissues and arterial plasma, respectively. The starting time of the linear section was fitted using an error criterion of 10%. Vt, Total distribution volume.


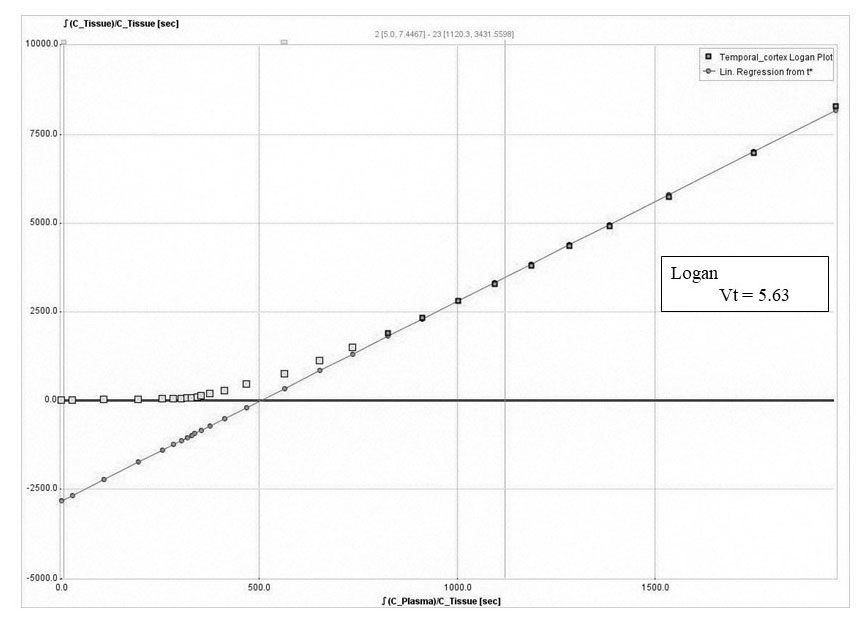


**Supplementary Fig. 4**. Correlations betweens Vts estimated by 1TCM, 2TCM, and Logan graphical analysis. Vt, Total distribution volume; 1TCM, a one-tissue compartment model; 2TCM, a two-tissue compartment model


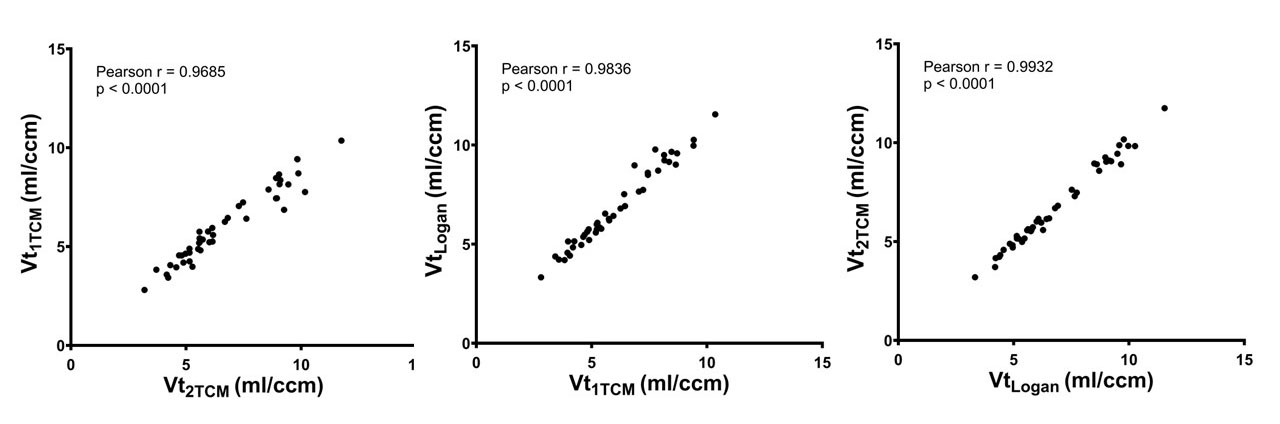


**Supplementary Table 1.** Comparison of χ2 criterion and AIC of the 1TCM and the 2TCM (n = 3)

|  | χ^2^  (mean) | | AIC (mean) | |
| --- | --- | --- | --- | --- |
|  | 1TCM | 2TCM | 1TCM | 2TCM |
| Cerebral deep white matter | 11.74 | 6.24 | 72.82 | 54.08 |
| Pallidum | 9.76 | 5.13 | 67.25 | 50.29 |
| Caudate | 5.84 | 4.14 | 53.55 | 46.08 |
| Thalamus | 10.74 | 5.66 | 70.82 | 54.61 |
| Cingulate cortex | 13.16 | 7.54 | 76.15 | 62.43 |
| Insular cortex | 13.39 | 8.12 | 76.89 | 64.80 |
| Parietal cortex | 12.25 | 6.80 | 74.15 | 59.24 |
| Amygdala | 10.76 | 7.97 | 69.42 | 62.50 |
| Temporal cortex | 11.30 | 6.51 | 71.52 | 57.35 |
| Frontal cortex | 12.52 | 6.83 | 74.73 | 59.13 |
| Occipital cortex | 13.34 | 7.41 | 76.28 | 60.94 |
| Putamen | 8.66 | 4.56 | 63.54 | 47.23 |
| Cerebellar cortex | 17.07 | 11.54 | 83.27 | 71.84 |
| Cerebellar white matter | 17.63 | 10.28 | 84.75 | 70.40 |
| Subcortical white matter | 12.48 | 8.20 | 74.28 | 61.78 |
| 1TCM, a one-tissue compartment model; 2TCM, a two-tissue compartment model; AIC, Akaike's information criterion | | | | |

**Supplementary Table 2.** Total distribution volumes estimated by the Logan graphical method (n =3)

|  |  | Vt (ml/ccm) | |
| --- | --- | --- | --- |
|  |  | mean | SD |
| Logan | Cerebral deep white matter | 5.81 | 0.57 |
|  | Pallidum | 9.38 | 0.29 |
|  | Caudate | 8.91 | 0.31 |
|  | Thalamus | 8.83 | 1.12 |
|  | Cingulate cortex | 6.88 | 0.71 |
|  | Insular cortex | 6.84 | 0.64 |
|  | Parietal cortex | 5.18 | 0.49 |
|  | Amygdala | 10.30 | 0.88 |
|  | Temporal cortex | 5.96 | 0.34 |
|  | Frontal cortex | 5.54 | 0.56 |
|  | Occipital cortex | 4.71 | 0.35 |
|  | Putamen | 9.24 | 0.56 |
|  | Cerebellar cortex | 3.98 | 0.47 |
|  | Cerebellar white matter | 5.07 | 0.49 |
|  | Subcortical white matter | 5.72 | 0.46 |
| Vt, Total distribution volume; SD, standard deviation | | | |

**Supplementary Table 3**. SUVs and SUVR-1 referenced various brain regions (n =6)

| Time frame: 30 to 50 min after [^18^F]SMBT-1 injection | | | | | | | | | | |
| --- | --- | --- | --- | --- | --- | --- | --- | --- | --- | --- |
|  | SUV (g/ml) | | SUVR-1 | | | | | | | |
| Reference region |  |  | Cerebral deep WM | | Subcortical WM | | Cerebellar CTX | | Cerebellar WM | |
|  | mean | SD | mean | SD | mean | SD | Mean | SD | mean | SD |
| Cerebral deep white matter | 1.70 | 0.43 | 0.00 | 0.00 | -0.07 | 0.06 | 0.30 | 0.16 | 0.05 | 0.09 |
| Pallidum | 3.13 | 0.56 | 0.89 | 0.18 | 0.76 | 0.22 | 1.46 | 0.40 | 0.99 | 0.24 |
| Caudate | 2.41 | 0.34 | 0.47 | 0.23 | 0.37 | 0.24 | 0.91 | 0.38 | 0.54 | 0.25 |
| Thalamus | 3.02 | 0.67 | 0.80 | 0.09 | 0.67 | 0.12 | 1.33 | 0.27 | 0.88 | 0.15 |
| Cingulate cortex | 2.28 | 0.67 | 0.33 | 0.09 | 0.23 | 0.06 | 0.72 | 0.17 | 0.40 | 0.13 |
| Insular cortex | 2.00 | 0.49 | 0.18 | 0.09 | 0.09 | 0.04 | 0.52 | 0.14 | 0.23 | 0.09 |
| Parietal cortex | 1.82 | 0.44 | 0.08 | 0.10 | 0.00 | 0.04 | 0.38 | 0.09 | 0.12 | 0.07 |
| Amygdala | 2.54 | 0.61 | 0.51 | 0.13 | 0.40 | 0.13 | 0.96 | 0.32 | 0.59 | 0.19 |
| Temporal cortex | 1.83 | 0.49 | 0.07 | 0.07 | -0.01 | 0.02 | 0.39 | 0.13 | 0.12 | 0.09 |
| Frontal cortex | 1.75 | 0.46 | 0.03 | 0.10 | -0.05 | 0.05 | 0.32 | 0.10 | 0.07 | 0.08 |
| Occipital cortex | 1.58 | 0.36 | -0.06 | 0.09 | -0.13 | 0.04 | 0.21 | 0.07 | -0.02 | 0.06 |
| Putamen | 2.81 | 0.53 | 0.69 | 0.17 | 0.57 | 0.16 | 1.20 | 0.31 | 0.78 | 0.19 |
| Cerebellar cortex | 1.33 | 0.35 | -0.22 | 0.09 | -0.28 | 0.06 | 0.00 | 0.00 | -0.19 | 0.04 |
| Cerebellar white matter | 1.62 | 0.40 | -0.04 | 0.08 | -0.11 | 0.05 | 0.23 | 0.07 | 0.00 | 0.00 |
| Subcortical white matter | 1.83 | 0.47 | 1.08 | 0.06 | 0.00 | 0.00 | 0.39 | 0.11 | 0.13 | 0.07 |
| Time frame: 50 to 70 min after [^18^F]SMBT-1 injection | | | | | | | | | | |
|  | SUV (g/ml) | | SUVR-1 | | | | | | | |
| Reference region |  | | Cerebral deep WM | | Subcortical WM | | Cerebellar CTX | | Cerebellar WM | |
|  | mean | SD | mean | SD | mean | SD | Mean | SD | mean | SD |
| Cerebral deep white matter | 1.55 | 0.26 | 0.00 | 0.00 | -0.01 | 0.03 | 0.54 | 0.21 | 0.14 | 0.11 |
| Pallidum | 2.87 | 0.45 | 0.87 | 0.17 | 0.84 | 0.15 | 1.86 | 0.37 | 1.13 | 0.27 |
| Caudate | 2.29 | 0.30 | 0.50 | 0.15 | 0.48 | 0.13 | 1.30 | 0.36 | 0.71 | 0.25 |
| Thalamus | 2.70 | 0.58 | 0.74 | 0.14 | 0.72 | 0.16 | 1.68 | 0.46 | 0.99 | 0.30 |
| Cingulate cortex | 1.95 | 0.35 | 0.26 | 0.03 | 0.24 | 0.04 | 0.94 | 0.29 | 0.44 | 0.16 |
| Insular cortex | 1.74 | 0.24 | 0.13 | 0.06 | 0.12 | 0.03 | 0.74 | 0.23 | 0.29 | 0.13 |
| Parietal cortex | 1.47 | 0.23 | -0.05 | 0.05 | -0.06 | 0.04 | 0.46 | 0.16 | 0.08 | 0.10 |
| Amygdala | 2.41 | 0.35 | 0.57 | 0.16 | 0.55 | 0.13 | 1.42 | 0.45 | 0.79 | 0.27 |
| Temporal cortex | 1.59 | 0.22 | 0.04 | 0.06 | 0.02 | 0.04 | 0.60 | 0.25 | 0.18 | 0.14 |
| Frontal cortex | 1.48 | 0.23 | -0.04 | 0.05 | -0.05 | 0.03 | 0.47 | 0.19 | 0.09 | 0.11 |
| Occipital cortex | 1.25 | 0.17 | -0.18 | 0.08 | -0.19 | 0.06 | 0.25 | 0.10 | -0.07 | 0.08 |
| Putamen | 2.53 | 0.33 | 0.65 | 0.13 | 0.63 | 0.10 | 1.54 | 0.37 | 0.89 | 0.24 |
| Cerebellar cortex | 1.02 | 0.19 | -0.34 | 0.09 | -0.35 | 0.08 | 0.00 | 0.00 | -0.25 | 0.05 |
| Cerebellar white matter | 1.37 | 0.26 | -0.11 | 0.09 | -0.13 | 0.08 | 0.35 | 0.08 | 0.00 | 0.00 |
| Subcortical white matter | 1.56 | 0.24 | 0.01 | 0.03 | 0.00 | 0.00 | 0.56 | 0.19 | 0.15 | 0.10 |
| Time frame: 70 to 90 min after [^18^F]SMBT-1 injection | | | | | | | | | | |
|  | SUV (g/ml) | | SUVR-1 | | | | | | | |
| Reference region |  | | Cerebral deep WM | | Subcortical WM | | Cerebellar CTX | | Cerebellar WM | |
|  | mean | SD | mean | SD | mean | SD | Mean | SD | mean | SD |
| Cerebral deep white matter | 1.24 | 0.24 | 0.00 | 0.00 | 0.04 | 0.03 | 0.68 | 0.22 | 0.18 | 0.12 |
| Pallidum | 2.48 | 0.59 | 0.98 | 0.15 | 1.05 | 0.18 | 2.34 | 0.59 | 1.35 | 0.37 |
| Caudate | 1.95 | 0.43 | 0.56 | 0.10 | 0.62 | 0.11 | 1.63 | 0.43 | 0.85 | 0.27 |
| Thalamus | 2.18 | 0.57 | 0.74 | 0.12 | 0.80 | 0.17 | 1.94 | 0.55 | 1.06 | 0.34 |
| Cingulate cortex | 1.48 | 0.32 | 0.19 | 0.03 | 0.23 | 0.04 | 1.00 | 0.30 | 0.41 | 0.16 |
| Insular cortex | 1.33 | 0.25 | 0.07 | 0.05 | 0.11 | 0.03 | 0.80 | 0.22 | 0.27 | 0.12 |
| Parietal cortex | 1.11 | 0.22 | -0.11 | 0.03 | -0.07 | 0.03 | 0.50 | 0.19 | 0.06 | 0.10 |
| Amygdala | 1.97 | 0.40 | 0.58 | 0.15 | 0.64 | 0.13 | 1.67 | 0.44 | 0.87 | 0.27 |
| Temporal cortex | 1.22 | 0.23 | -0.01 | 0.05 | 0.02 | 0.04 | 0.66 | 0.25 | 0.17 | 0.14 |
| Frontal cortex | 1.10 | 0.21 | -0.12 | 0.05 | -0.09 | 0.03 | 0.48 | 0.17 | 0.04 | 0.09 |
| Occipital cortex | 0.98 | 0.18 | -0.21 | 0.04 | -0.18 | 0.04 | 0.32 | 0.14 | -0.07 | 0.08 |
| Putamen | 2.04 | 0.41 | 0.64 | 0.10 | 0.69 | 0.10 | 1.75 | 0.41 | 0.94 | 0.25 |
| Cerebellar cortex | 0.74 | 0.12 | -0.40 | 0.08 | -0.37 | 0.08 | 0.00 | 0.00 | -0.29 | 0.04 |
| Cerebellar white matter | 1.05 | 0.16 | -0.15 | 0.09 | -0.12 | 0.09 | 0.42 | 0.07 | 0.00 | 0.00 |
| Subcortical white matter | 1.20 | 0.22 | -0.03 | 0.03 | 0.00 | 0.00 | 0.62 | 0.20 | 0.14 | 0.11 |
| SUV, standardized uptake value; SUVR, standardized uptake value ratio; Cerebral deep WM, cerebral deep white matter; Subcortical WM, subcortical white matter; Cerebellar CTX, cerebellar cortex; Cerebellar WM, cerebellar white matter; SD, standard deviation. | | | | | | | | | | |

**Supplementary Table 4**. Parameter estimation results from compartmental model analysis with vB fixed at 3%, 5%, and not fixed. (frontal and temporal cortices of one subject)

| Model | Brain regions | vB | Parameters | |  |  |  |  |
| --- | --- | --- | --- | --- | --- | --- | --- | --- |
|  |  |  | vB | K1 | k2 | Vt |  |  |
| 1TCM | Frontal cortex | fixed to 3% | 0.030 | 0.256 | 0.044 | 5.762 |  |  |
|  |  | fixed to 5% | 0.050 | 0.253 | 0.043 | 5.916 |  |  |
|  |  | not fixed | 0.096 | 0.246 | 0.039 | 6.302 |  |  |
|  |  |  |  |  |  |  |  |  |
|  | Temporal cortex | fixed to 3% | 0.030 | 0.228 | 0.038 | 5.942 |  |  |
|  |  | fixed to 5% | 0.050 | 0.225 | 0.037 | 6.111 |  |  |
|  |  | not fixed | 0.086 | 0.220 | 0.034 | 6.442 |  |  |
|  |  |  |  |  |  |  |  |  |
| Model | Brain regions | vB | Parameters | |  |  |  |  |
|  |  |  | vB | K1 | k2 | k3 | k4 | Vt |
| 2TCM | Frontal cortex | fixed to 3% | 0.030 | 0.915 | 8.000 | 2.630 | 0.051 | 5.956 |
|  |  | fixed to 5% | 0.050 | 0.296 | 0.221 | 0.417 | 0.119 | 6.024 |
|  |  | not fixed | 0.094 | 0.252 | 0.046 | 0.004 | 0.000 | 8.590 x 10^97^ |
|  |  |  |  |  |  |  |  |  |
|  | Temporal cortex | fixed to 3% | 0.030 | 0.780 | 8.000 | 2.859 | 0.046 | 6.141 |
|  |  | fixed to 5% | 0.050 | 0.250 | 0.116 | 0.175 | 0.092 | 6.276 |
|  |  | not fixed | 0.083 | 0.226 | 0.041 | 0.005 | 0.004 | 13.060 |
| vB, blood volume; 1TCM, a one-tissue compartment model; 2TCM, a two-tissue compartment model; Vt, Total distribution volume | | | | | | | | |
